# Supplementary material for: Identification of photoperiod-induced specific miRNAs in the adrenal glands of Sunite sheep (Ovis aries)
Source: Front Vet Sci. 2022 Jul 22;9:888207. doi: 10.3389/fvets.2022.888207 (PMC9354845; doi:10.3389/fvets.2022.888207)
Supplement: Supplementary file 1 [file Data_Sheet_1.ZIP › Supplementary file/miRDeep2 file.zip!/miRDeep2 file/Know_Structure/SPLP42A3/oar-mir-23b.pdf]

miRBase precursor : oar-mir-23b
Total read count : 8958
oar-miR-23b read count : 8939
remaining reads : 19

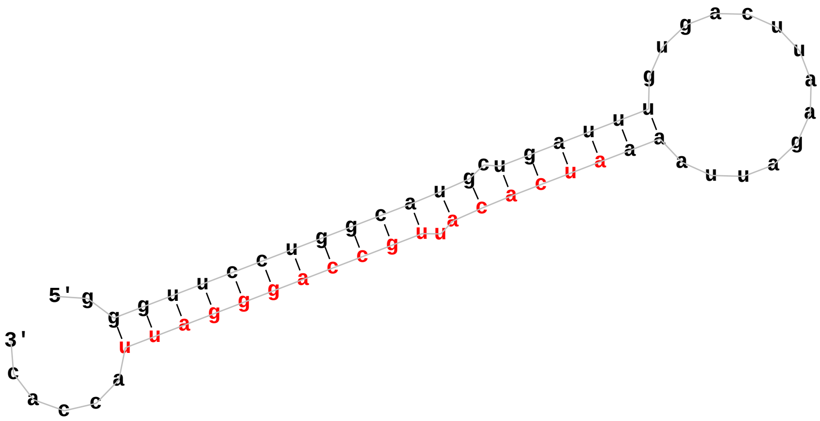

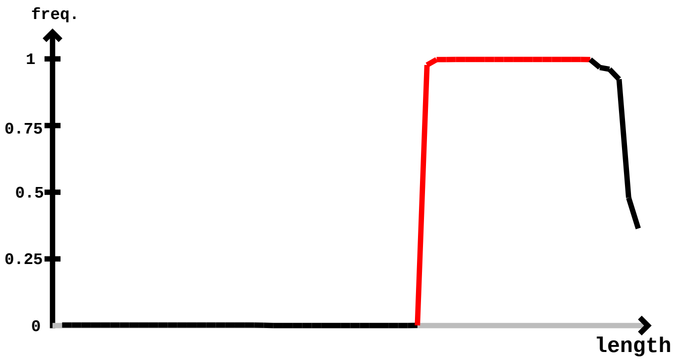


```
                                    oar-miR-23b
5'- ggguuccuggcaugcugauuugugacuuaagauuaaaaucacauugccagggauuaccac   -3'    exp
    ((((((((((((((.((((((..............)))))))).))))))))))..))..    reads     mm        sample
    ggguuccuggcaugcugau.........................................    1         0         seq
    ggguuccuggcaugcugauu........................................    7         0         seq
    ggguuccuggcaugcugauuu.......................................    10        0         seq
    ....................ugugacuuaagauuaaaa......................    1         0         seq
    ....................................aaucacauugccagggauu.....    2         0         seq
    ....................................aaucacauugccagggauuac...    1         0         seq
    ....................................aaucacauugccagggauuacc..    2         0         seq
    .....................................aucacauugccagggau......    3         0         seq
    .....................................aucacauugccagggaC......    1         1         seq
    .....................................aucacauugcUagggauu.....    1         1         seq
    .....................................aucacauugccagggauu.....    263       0         seq
    .....................................aucacauugccagggauua....    58        0         seq
    .....................................aucacauugccagggauuac...    329       0         seq
    .....................................aucacauugccagggauuacc..    3927      0         seq
    .....................................aucacauugccagggauuacca.    1015      0         seq
    .....................................aucacauugccagggauuaccac    3149      0         seq
    ......................................ucacauugccagggauua....    1         0         seq
    ......................................ucacauugccagggauuac...    5         0         seq
    ......................................ucacauugccagggauuacc..    50        0         seq
    ......................................ucacauugccagggauuacca.    16        0         seq
    ......................................ucacauugccagggauuaccac    112       0         seq
    .......................................cacauugccagggauuacc..    1         0         seq
    ........................................acauugccagggauuacc..    2         0         seq
    ........................................acauugccagggauuacca.    1         0         seq
```
